# Depth from a Motion Algorithm and a Hardware Architecture for Smart Cameras

**DOI:** 10.3390/s19010053

**Published:** 2018-12-23

**Authors:** Abiel Aguilar-González, Miguel Arias-Estrada, François Berry

**Affiliations:** 1 Instituto Nacional de Astrofísica, Óptica y Electrónica (INAOE), Tonantzintla 72840, Mexico; ariasmo@inaoep.mx; 2 Institut Pascal, Université Clermont Auvergne (UCA), 63178 Clermont-Ferrand, France; francois.berry@uca.fr

**Keywords:** depth estimation, monocular systems, optical flow, smart cameras, FPGA (Field Programmable Gate Array)

## Abstract

Applications such as autonomous navigation, robot vision, and autonomous flying require depth map information of a scene. Depth can be estimated by using a single moving camera (depth from motion). However, the traditional depth from motion algorithms have low processing speeds and high hardware requirements that limit the embedded capabilities. In this work, we propose a hardware architecture for depth from motion that consists of a flow/depth transformation and a new optical flow algorithm. Our optical flow formulation consists in an extension of the stereo matching problem. A pixel-parallel/window-parallel approach where a correlation function based on the sum of absolute difference (SAD) computes the optical flow is proposed. Further, in order to improve the SAD, the curl of the intensity gradient as a preprocessing step is proposed. Experimental results demonstrated that it is possible to reach higher accuracy (90% of accuracy) compared with previous Field Programmable Gate Array (FPGA)-based optical flow algorithms. For the depth estimation, our algorithm delivers dense maps with motion and depth information on all image pixels, with a processing speed up to 128 times faster than that of previous work, making it possible to achieve high performance in the context of embedded applications.

## 1. Introduction

Smart cameras are machine vision systems which, in addition to image capture circuitry, are capable of extracting application-specific information from captured images. For example, for video surveillance, image processing algorithms implemented inside the camera fabric can detect and track pedestrians [1], but for a robotic application, computer vision algorithms could estimate the system’s egomotion [2]. In recent years, advances in embedded vision systems such as progress in microprocessor power and FPGA technology led to the creation of compact smart cameras with increased performance for real world applications [3,4,5,6]. As a result, in current embedded applications, image processing algorithms inside the smart camera’s fabric deliver an efficient on-board solution for motion detection [7], object detection/tracking [8,9], inspection and surveillance [10], human behavior recognition [11], etc. Computer vision algorithms can also be frequently used by smart cameras since they are the basis of several applications (automatic inspection, controlling processes, detecting events, modeling objects or environments, navigation, and so on). Unfortunately, mathematical formulation of computer vision algorithms is not compliant with the hardware technologies (FPGA/CUDA) often used in smart cameras. In this work, we are interested in depth estimation from monocular sequences in the context of a smart camera because depth is the basis to obtain useful scene abstractions, for example, 3D reconstructions of the world and camera egomotion.

### 1.1. Depth Estimation from Monocular Sequences

In several applications, such as autonomous navigation [12], robot vision and surveillance [1], and autonomous flying [13], there is a need for determining the depth map of the scene. Depth can be estimated by using stereo cameras [14], by changing focal length [15] or by employing a single moving camera [16]. In this work, we are interested in depth estimation from monocular sequences by using a single moving camera (depth from motion). This choice is motivated because monocular systems have higher efficiency compared with other approaches, simpler and more accurate than defocus techniques and, cheaper/smaller compared with stereo-based techniques. In monocular systems, depth information can be estimated based on two or multiple frames of a video sequence. For two frames, image information may not provide sufficient information for accurate depth estimation. The use of multiple frames improves the accuracy, reduces the influence of noise, and allows the extraction of additional information that cannot be recovered from just two frames, but the system complexity and computational cost is increased. In this work, we use information from two consecutive frames of the monocular sequence since our algorithm is focused on smart cameras, and in this context hardware resources are limited.

### 1.2. Motivation and Scope

In the last decade, several works have demonstrated that depth information is highly useful for embedded robotic applications [1,12,13]. Unfortunately, depth information estimation is a relatively complex task. In recent years, the most popular solution is the use of active vision to estimate depth information from a scene [17,18,19,20,21], i.e., LIDAR sensors or RGBD cameras that can deliver accurate depth maps in real time; however, they increase the system’s size and cost. In this work, we propose a new algorithm and an FPGA hardware architecture for depth estimation. First, a new optical flow algorithm estimates the motion (flow) at each point in the input image. A flow/depth transformation then computes the depth in the scene. For the optical flow algorithm, an extension of the stereo matching problem is proposed. A pixel-parallel/window-parallel approach where a sum of absolute difference (SAD) computes the optical flow is implemented. Further, in order to improve the SAD, we propose the curl of the intensity gradient as a preprocessing step. For the depth estimation proposes, we introduce a flow/depth transformation inspired by epipolar geometry.

## 2. Related Work

In previous work, depth estimation is often estimated by using a single moving camera. This approach is called depth from motion and consists in computing the depth from the pixel velocities inside the scene (optical flow); i.e., optical flow is the basis for depth from motion.

### 2.1. FPGA Architectures for Optical Flow

In Ref. [22], a hardware implementation of a high complexity algorithm to estimate the optical flow from image sequences in real time is presented. In order to fulfil with the architectural limitations, the original gradient-based optical flow was modified (using a smoothness constraint for decreasing iterations). The developed architecture can estimate the optical flow in real time and can be constructed with FPGA or ASIC devices. However, due to the mathematical limitations of the CPU formulation (complex/iterative operations), speed processing is low, compared with other FPGA-based architectures for real-time image processing [23,24]. In Ref. [25], a pipelined optical-flow processing system that works as a virtual motion sensor is described. The proposed approach consists of several spatial and temporal filters (Gaussian and gradient spatial filters and IIR temporal filter) implemented in cascade. The proposed algorithm was implemented in an FPGA device, enabling the easy change of the configuration parameters to adapt the sensor to different speeds, light conditions, and other environmental factors. This makes possible the implementation of an FPGA-based smart camera for optical flow. In general, the proposed architecture reaches a reasonable level of hardware resource usage, but accuracy and processing speed is low (lower than 7 fps for 640 × 480 image resolution). In Ref. [26], a tensor-based optical flow algorithm is presented. This algorithm was developed and implemented using FPGA technology. Experimental results demonstrated high accuracy compared with previously FPGA-based algorithms for optical flow. In addition, the proposed design can process 640 × 480 images at 64 fps with a relatively low resource requirement, making it easier to fit into small embedded systems. In Ref. [27], a highly parallel architecture for motion estimation is presented. The developed FPGA-architecture implements the Lucas and Kanade algorithm [28] with the multi-scale extension for the computation of large motion estimations in an FPGA. Although the proposed architecture reaches a low hardware requirement with a high processing speed, the use of a huge external memory capacity is needed. Further, due to the low hardware requirements, the accuracy is low (near 11% more error compared with the original CPU version of the Lukas and Kanade algorithm). Finally, in Ref. [29], an FPGA-based platform with the capability of calculating real-time optical flow at 127 frames per second for a 376 × 240 pixel resolution is presented. Radial undistortion, image rectification, disparity estimation, and optical flow calculation tasks are performed on a single FPGA without the need for external memory. Therefore, the platform is perfectly suited for mobile robots or embedded applications. Unfortunately, accuracy is low (qualitatively lower accuracy than CPU-based approaches).

### 2.2. Optical Flow Methods Based on Learning Techniques

There are some recent works that addresses the optical flow problem via learning techniques [30]. In 2015, Ref. [31] proposed the use of convolutional neuronal networks (CNNs) as an alternative framework to solve the optical flow estimation problem. Two different architectures were proposed and compared: a generic architecture and another one including a layer that correlates feature vectors at different image locations. Experimental results demonstrated a competitive accuracy at frame rates of 5–10 fps. On the other hand, in 2017, Ref. [32] developed a stacked architecture that includes a warping of the search image with intermediate optical flow. Further, in order to achieve high accuracy on small displacements, the authors introduced a sub-network specializing on small motions. Experimental results demonstrated that it is possible to reach an accuracy of more than 95%, decreasing the estimation error by more than 50%, compared with previous works.

## 3. The Proposed Algorithm

**Figure 1:**
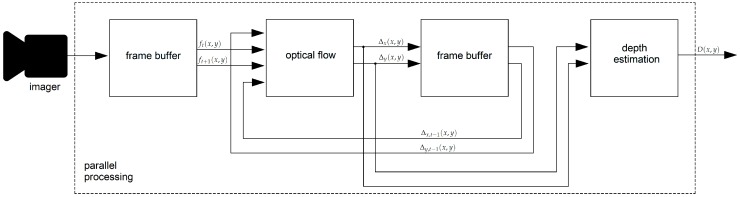
Block diagram of the proposed algorithm.

In Figure 1, an overview of our algorithm is shown. First, given an imager as sensor, two consecutive frames $\left(f_{t}\left(x,y\right),\;f_{t+1}\left(x,y\right)\right)$ are stored in local memory. Then, an optical flow algorithm computes 2D pixel displacements between $f_{t}\left(x,y\right)$ and $f_{t+1}\left(x,y\right)$. A dynamic template based on the optical flow previously computed $\left(\Delta_{x,t-1}\left(x,y\right),\;\Delta_{y,t-1}\left(x,y\right)\right)$ computes the search region size for the current optical flow. We then let the optical flow for the current frame be $\left(\Delta_{x}\left(x,y\right),\;\Delta_{y}\left(x,y\right)\right)$. The final step is depth estimation for all the pixels in the reference image D(x,y). In the following subsections, details about the proposed algorithm are presented.

### 3.1. Frame Buffer

The first step in our mathematical formulation is image storage. Considering that in most cases the imager provides data as a stream, some storage is required in order to have two consecutive frames available at the same time *t*. More information about the storage architecture is presented in Section 4.1. For mathematical formulation, we consider the first frame (frame at *t* time) as $f_{t}\left(x,y\right)$, while the second frame (frame at t+1 time) is $f_{t+1}\left(x,y\right)$.

### 3.2. Optical Flow

**Figure 2:**
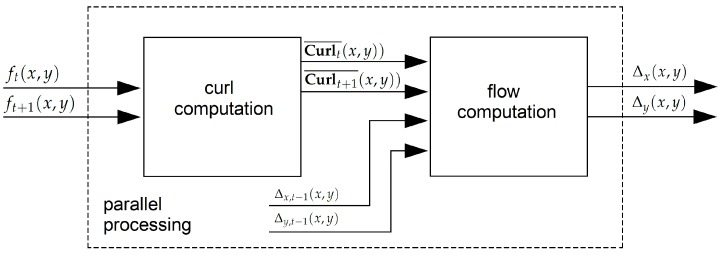
The optical flow step: first, curl images $\left(\bar{{\mathrm{Curl}}_{t}}\left(x,y\right)\right)$, $\left(\bar{{\mathrm{Curl}}_{t+1}}\left(x,y\right)\right)$ are computed. Then, given the curl images for two consecutive frames, pixels displacements $\Delta_{x}\left(x,y\right)$, $\Delta_{y}\left(x,y\right)$ (optical flow for all pixels in the reference image) are computed using a dynamic template based on the optical flow previously computed $\left(\Delta_{x,t-1}\left(x,y\right),\;\Delta_{y,t-1}\left(x,y\right)\right)$.

**Figure 3:**
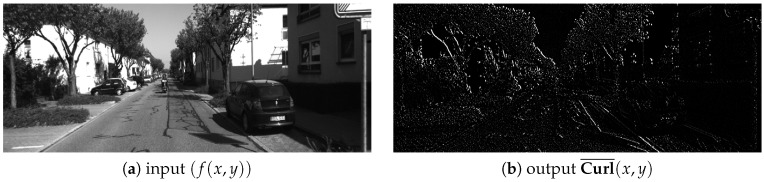
Curl computation example. Input image taken from the KITTI benchmark dataset [35].

**Figure 4:**
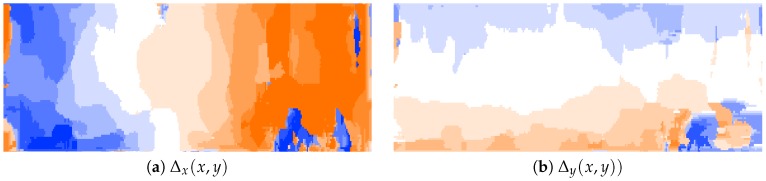
Optical flow example. Image codification as proposed in the Tsukuba benchmark dataset [36].

**Figure 5:**
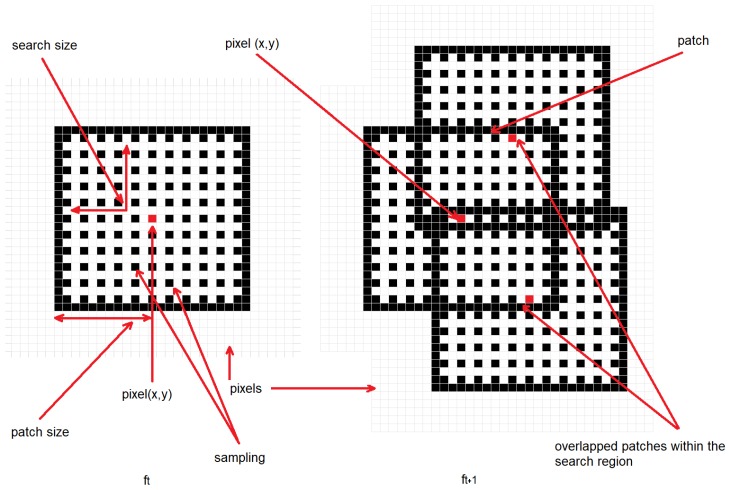
The proposed optical flow algorithm formulation: patch size = 10, search size = 10, and sampling value = 2. For each pixel in the reference image $f_{t}$, *n* overlapped regions are constructed in $f_{t+1}$, and the *n* region center that minimizes or maximizes any similarity metric is the tracked position (flow) of the pixel (x,y) at $f_{t+1}$.

In previous works, iterative algorithms, such as the Lucas Kanade [28] or the Horn–Schunck [33] algorithms, have been used in order to compute optical flow across video sequences; given dense optical flow, geometric methods allow one to compute the depth of the scene. However, these algorithms [28,33] have iterative operations that limit the performance for smart camera implementations. In order to avoid the iterative and convergence part of the traditional formulation, we replace that with a correlation metric implemented inside a pixel-parallel/window-parallel formulation. In Figure 2, an overview of our optical flow algorithm is shown. Let $(f_{t}\left(x,y\right),f_{t+1}\left(x,y\right)$ be two consecutive frames from a video sequence. The curl of the intensity gradient $\frac{df(x,y)}{dx}$ is computed (see Equation (1)), where ∇ is the **Del** operator. Let the curl be a vector operator that describes the infinitesimal rotation; then, at every pixel, the curl of that pixel is represented by a vector where attributes (length and direction) characterize the rotation at that point. In our case, we use only the norm of $\bar{\mathrm{Curl}}\left(x,y\right)$, as shown in Equation (2) and as illustrated in Figure 3. This operation increases the robustness under image degradations (color/texture repetition, illumination changes, and noise); therefore, simple similarity metrics [34] deliver accurate pixel tracking, simpler than previous tracking algorithms [28,33]. Then, using the curl images for two consecutive frames as inputs ($\bar{{\mathrm{Curl}}_{t}}\left(x,y\right)$ and $\bar{{\mathrm{Curl}}_{t+1}}\left(x,y\right)$), the dense optical flow ($\Delta_{x}\left(x,y\right),\;\Delta_{y}\left(x,y\right)$, illustrated in Figure 4) is computed, as shown in Figure 5. This process assumes that pixel displacements between frames is such that it contains an overlap on two successive “search regions.” A search region is defined as a patch around a pixel to track. Considering that, between $f_{t}$ and $f_{t+1}$, the image degradation is low, any similarity-based metric has to provide good accuracy. In our case, this similarity is calculated by a SAD. This process is defined in Equation (3), where *r* is the patch size (see Figure 5). $\left(\bar{{\mathrm{Curl}}_{t}}\left(x,y\right),\bar{{\mathrm{Curl}}_{t+1}}\left(x,y\right)\right)$ are curl images on two consecutive frames. x,y are the spatial coordinates of pixels in $f_{t}$, and a,b are the spatial coordinates within a search region constructed in $f_{t+1}$ (see Equations (4) and (5)), where $\Delta_{x(t-1)}^{\prime },\Delta_{y(t-1)}^{\prime }$ represent a dynamic search template, computed as shown in Section 3.3. *k* is the search size, and *s* is a sampling value defined by the user. Finally, the optical flow at the current time $\left(\Delta_{x}\left(x,y\right),\;\Delta_{y}\left(x,y\right)\right)$ is computed by Equation (6).

(1) $$\mathrm{Curl}\left(x,y\right)=\nabla \times \frac{df(x,y)}{dx}=\frac{\partial }{\partial y}\frac{\partial f(x,y)}{\partial x}-\frac{\partial }{\partial x}\frac{\partial f(x,y)}{\partial y}$$

(2) $$\bar{\mathrm{Curl}}\left(x,y\right)=\left|\frac{\partial }{\partial y}\frac{\partial f(x,y)}{\partial x}-\frac{\partial }{\partial x}\frac{\partial f(x,y)}{\partial y}\right|$$

where

$$\frac{\partial f(x,y)}{\partial x}=G_{x}\left(x,y\right)=f\left(x+1,y\right)-f\left(x-1,y\right)$$

$$\frac{\partial f(x,y)}{\partial y}=G_{y}\left(x,y\right)=f\left(x,y+1\right)-f\left(x,y-1\right)$$

$$\frac{\partial }{\partial y}\frac{\partial f(x,y)}{\partial x}=G_{x}\left(x,y+1\right)-G_{x}\left(x,y-1\right)$$

$$\frac{\partial }{\partial x}\frac{\partial f(x,y)}{\partial y}=G_{y}\left(x+1,y\right)-G_{y}\left(x-1,y\right)$$

(3) $$\begin{array}{c} \mathrm{SAD}\left(a,b\right)=\sum_{u=-r}^{u=r}\sum_{v=-r}^{v=r}|\bar{{\mathrm{Curl}}_{t}}\left(x+u,y+v\right)|-|\bar{{\mathrm{Curl}}_{t+1}}\left(x+u+a,y+v+b\right)| \end{array}$$

(4) $$\begin{array}{c} a=\Delta_{x(t-1)}^{\prime }\left(x,y\right)-k:s:\Delta_{x(t-1)}^{\prime }\left(x,y\right)+k \end{array}$$

(5) $$\begin{array}{c} b=\Delta_{y(t-1)}^{\prime }\left(x,y\right)-k:s:\Delta_{y(t-1)}^{\prime }\left(x,y\right)+k \end{array}$$

(6) $$\begin{array}{c} \left[\Delta_{x}\left(x,y\right),\Delta_{y}\left(x,y\right)\right]={\arg\;\min}_{\left(a,b\right)}SAD\left(a,b\right). \end{array}$$

### 3.3. Search Template

In optical flow, the search window size defines the maximum allowed motion to be detected in the sequence, see Figure 4. In general, let *p* be a pixel in the reference image $\left(f_{t}\right)$, whose 2D spatial location is defined as $\left(x_{t},y_{t}\right)$, the same pixel in the tracked image $\left(f_{t+1}\right)$ has to satisfy $x_{t+1}\in x_{t}-k:1:x+k$, $y_{t+1}\in y-k:1:y_{t}+k$, where *k* is the search size for the tracking step. In practice, large search region sizes increase tracking performance since feature tracking could be carried out in both slow and fast camera movements. However, large search sizes decrease the accuracy, i.e., if the search region size is equal to 1, then $x_{t+1}\in x_{t}-1:1:x_{t}+1$, $y_{t+1}\in y_{t}-1:1:y_{t}+1$, so there are nine possible candidates for the tracking step and the mistake possibility is equal to 8, this considering that camera movement is slow and therefore pixel displacements between images are close to zero. In other scenarios, if the search region size is equal to 10, then $x_{t+1}\in x_{t}-10:1:x_{t}+10$, $y_{t+1}\in y_{t}-10:1:y_{t}+10$, so there are 100 possible candidates for the tracking step, and the mistake possibility is equal to 99. In our work, we propose using the feedback of the previous optical flow step as a dynamic search size for the current step. Therefore, if camera movement in t−1 is slow, small search sizes closer to the pixels being tracked $\left(x_{t},y_{t}\right)$ are used. On the other hand, given fast camera movements, small search sizes far from the pixels being tracked are used. This makes the tracking step compute accurate results without outliers; furthermore, the use of small search sizes decreases the computational resources usage. For practical purposes, we use a search region size equal to 10 since it provides a good tradeoff between robustness/accuracy and computational resources. Therefore, let $\Delta_{x,t-1}\left(x,y\right),\;\Delta_{y,t-1}\left(x,y\right)$ be the optical flow at time t−1, the search template for the current time is computed as shown in Equations (7) and (8), where *k* is the template size.

(7) $$\begin{array}{c} \Delta_{x}^{\prime }\left(x+u,y+v\right)=\sum_{u=-k,v=-k}^{u=k,v=k}\left(\mathrm{mean}\sum_{u=-k,v=-k}^{u=k,v=k}\Delta_{x,t-1}\left(x,y\right)\right) \end{array}$$

(8) $$\begin{array}{c} \Delta_{y}^{\prime }\left(x+u,y+v\right)=\sum_{u=-k,v=-k}^{u=k,v=k}\left(\mathrm{mean}\sum_{u=-k,v=-k}^{u=k,v=k}\Delta_{y,t-1}\left(x,y\right)\right) \end{array}$$

### 3.4. Depth Estimation

**Figure 6:**
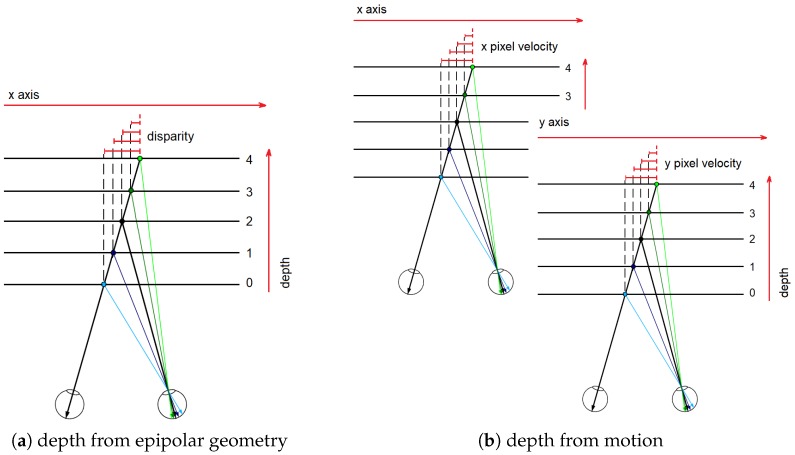
(**a**) Epipolar geometry: depth in the scene is proportional to the disparity value, i.e., far objects have low disparity values, while closer objects are associated with high disparity values. To compute the disparity map (disparities for all pixels in the image) a stereo pair (two images with epipolar geometry) are needed. (**b**) Single moving camera: in this work we suppose that depth in the scene is proportional to the pixel velocity across the time. To compute the pixel velocity, optical flow across two consecutive frames has to be computed.

In previous works, it was demonstrated that monocular image sequences provide only partial information about a scene due to the computation of relative depth, the unknown scale factor, etc. [37]. In order to recover the depth in the scene, it is necessary to have assumptions about the scene and its 2-D images. In this work, we assume that the environment within the scene is rigid. Thus, given the optical flow of the scene (which represents pixel velocity across time), we suppose that the depth in the scene is proportional to the pixel velocity; i.e., far objects have to be associated with a low velocity value, while closer objects are associated with high velocity values. This could be considered an extension of the epipolar geometry in which disparities values are proportional to the depth in the scene, as shown in Figure 6.

**Figure 7:**
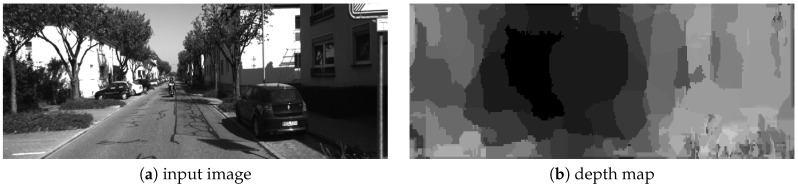
Depth estimation using the proposed algorithm.

Therefore, let $\Delta_{x}\left(x,y\right)$ and $\Delta_{y}\left(x,y\right)$ be the optical flow (pixel velocity) at *t* time. The depth in the scene depth(x,y) is computed as proposed in Equation (9), where depth(x,y) is the norm of the optical flow. In Figure 7, an example of depth map computed by the proposed approach is shown.

(9) $$\begin{array}{c} \mathrm{depth}\left(x,y\right)=∥\left[\Delta_{x}\left(x,y\right),\Delta_{y}\left(x,y\right)\right]∥=\sqrt{\Delta_{x}{\left(x,y\right)}^{2}+\Delta_{y}{\left(x,y\right)}^{2}}. \end{array}$$

## 4. The FPGA Architecture

**Figure 8:**
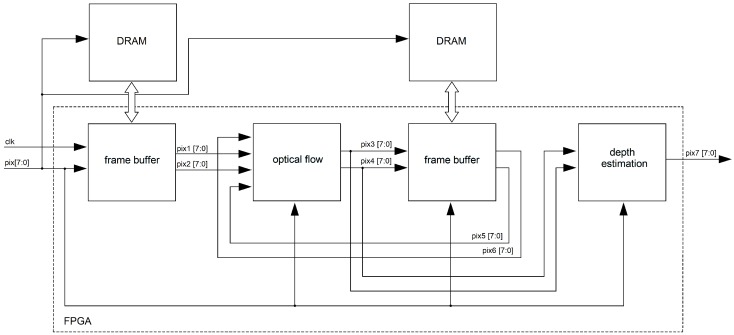
FPGA architecture for the proposed algorithm.

In Figure 8, an overview of the FPGA architecture for the proposed algorithm is shown. The architecture is centered on an FPGA implementation where all recursive/parallelizable operations are accelerated in the FPGA fabric. First, the “frame buffer” unit reads the pixel stream (pix [7:0]) delivered by the imager. In this block, frames captured by the imager are fed to/from an external DRAM memory and deliver pixel streams for two consecutive frames in parallel (pix1 [7:0], pix2 [7:0]). “Circular buffers” implemented inside the “optical flow” unit are used to hold local sections of the frames that are being processed and allow for local parallel access that facilitates parallel processing. Finally, optical flow streams (pix3 [7:0], pix4 [7:0]) are used to compute the depth in the scene (pix7 [7:0]). In order to hold optical flow previously computed (which are used for the dynamic search template computation), a second “frame buffer” is used. In the following subsections, details about the algorithm parallelization are shown.

### 4.1. Frame Buffer

Images from the image sensor are stored in an external DRAM that holds an entire frame from the sequence, and later the DRAM data are read by the FPGA to cache pixel flow of the stored frame into circular buffers. In order to deliver two consecutive frames in parallel, two DRAM chips in switching mode are used, i.e.,

1. $t_{1}$: DRAM 1 in write mode (storing Frame 1), DRAM 2 in read mode (invalid values), Frame 1 at Output 1, invalid values at Output 2.
2. $t_{2}$: DRAM 1 in read mode (reading Frame 1), DRAM 2 in write mode (storing Frame 1), Frame 1 at Output 2, Frame 1 at Output 2.
3. $t_{3}$: DRAM 1 in write mode (storing Frame 3), DRAM 2 in read mode (reading Frame 2), Frame 3 at Output 2, Frame 2 at Output 2 and so on.

**Figure 9:**
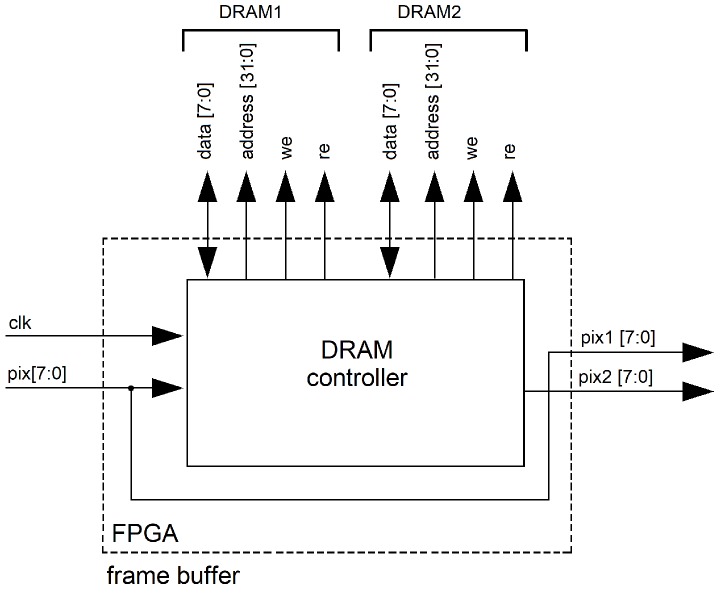
FPGA architecture for the “frame buffer” unit. Two external memories configured in switching mode makes it possible to store the current frame (time *t*) into a DRAM configured in write mode, while another DRAM (in read mode) deliver pixel flow for a previous frame (frame at time t−1).

In Figure 9, an overview of the “frame buffer” unit is shown. The current pixel stream (pix [7:0]) is mapped at Output 1 (pix1 [7:0]), while Output 2 (pix2 [7:0]) delivers pixel flow for a previous frame. For the external DRAM control, data [7:0] are mapped with the read/write pixel stream, address [31:0] manages the physical location inside the memory, and the “we” and “re” signals enable the write/read process respectively, as shown in Figure 9.

### 4.2. Optical Flow

**Figure 10:**
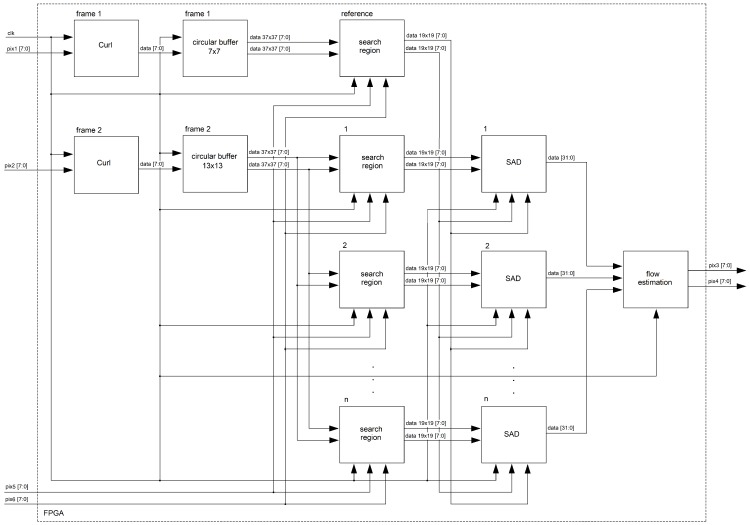
FPGA architecture for the optical flow estimation.

For the “optical flow” unit, we consider that the flow estimation problem can be a generalization of the dense matching problem; i.e., stereo matching algorithms track (searching along the horizontal axis around the search image) all pixels in the reference image. Optical flow aims to track all pixels between two consecutive frames from a video sequence (searching around spatial coordinates of the pixels in the search image). It is then possible to extend previous stereo matching FPGA architectures to our application domain. In this work, we extended the FPGA architecture presented in [24], since it has low hardware requirements and a high level of parallelism. In Figure 10, the developed architecture is shown. First, the “curl” units deliver curl images in parallel (see Equation (2)). More details about the FPGA architecture of this unit are shown in Section 4.2.2. The “circular buffer” units are responsible for data transfers in segments of the image (usually several rows of pixels). Therefore, the core of the FPGA architecture are the circular buffers attached to the local processors that can hold temporarily as cache, for image sections from two frames, and that can deliver parallel data to the processors. More details about the FPGA architecture of this unit are shown in Section 4.2.1. Then, given the optical flow previously computed, 121 search regions are constructed in parallel (see Figure 5 and Equations (4) and (5)). For our implementation, the search region size is equal to 10, so the center of the search regions are all the sampled pixels within the reference region. Given the reference region in $f_{t}\left(x,y\right)$ and 121 search regions in $f_{t+1}\left(x,y\right)$, search regions are compared with the reference region (Equation (3)) in parallel. For that, a pixel-parallel/window-parallel scheme is implemented. Finally, in the “flow estimation” unit, a multiplexer tree can determine the a,b indices that minimize Equation (3) and therefore, using Equation (6), the optical flow for all pixels in the reference image.

#### 4.2.1. Circular Buffer

**Figure 11:**
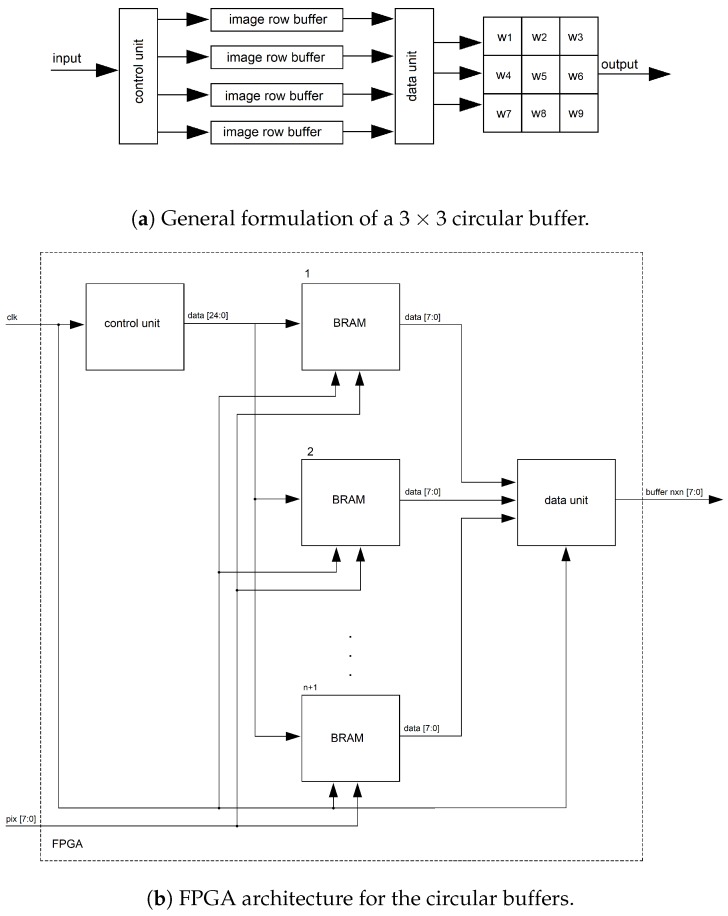
The circular buffers architecture. For an n×n patch, a shift mechanism “control” unit manages the read/write addresses of n+1 BRAMs. In this formulation, *n* BRAMs are in read mode, and one BRAM is in write mode in each clock cycle. The n×n buffer then delivers logic registers with all pixels within the patch in parallel.

In Ref. [23], we proposed circular buffer schema in which input data from the previous *n* rows of an image can be stored using memory buffers (block RAMs/BRAMs) until the moment when an n×n neighborhood is scanned along subsequent rows. In this work, we follow a similar approach to achieve high data reuse and a high level of parallelism. Our algorithm is then processed in modules where all image patches can be read in parallel. First, a shift mechanism “control” unit manages the read/write addresses of n+1 BRAMs. In this formulation, *n* BRAMs are in read mode, and one BRAM is in write mode in each clock cycle. Data inside the read-mode BRAMs can then be accessed in parallel, and each pixel within an n×n region is delivered in parallel with an n×n buffer, as shown in Figure 11, where the “control” unit delivers control data (address and read/write enable) for the BRAM modules, and one entire row is stored in each BRAM. Finally the “data” unit delivers n×n pixels in parallel. In our implementation, there is one circular buffer of 13 × 13 pixels/bytes, one circular buffer of 17 × 17, and two circular buffers of 3 × 3. For more details, see Ref. [23].

#### 4.2.2. Curl Estimation

**Figure 12:**
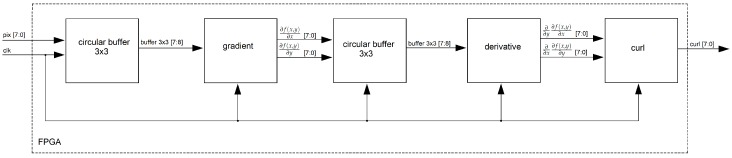
FPGA architecture for the “curl” unit.

In Figure 12, the curl architecture is shown. First, one “circular buffer” holds three rows of the frame being processed and allows for local parallel access of a 3×3 patch that facilitates parallel processing. Then, image gradients ($\frac{\partial f(x,y)}{\partial x},\;\frac{\partial f(x,y)}{\partial y}$) are computed. Another “circular buffer” holds three rows of the gradient image previously computed and delivers a 3×3 patch for the next step. Second derivatives ($\frac{\partial }{\partial y}\frac{\partial f(x,y)}{\partial x},\;\frac{\partial }{\partial x}\frac{\partial f(x,y)}{\partial y}$) are computed inside the “derivative” unit. Finally, the curl of the input image is computed by the “curl” unit.

### 4.3. Depth Estimation

**Figure 13:**
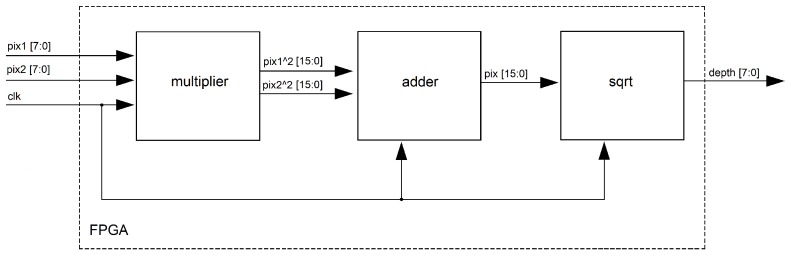
FPGA architecture for the “depth estimation” unit.

In Figure 13, the depth estimation architecture is shown. Let “pix1 [7;0]”, “pix2 [7:0]” be the pixel stream for the optical flow at the current frame (Equation (6)); first, the “multiplier” unit computes the square value of the input data. Then, the “adder” unit carries out the addition process for both components $\left(\Delta_{x}^{2},\Delta_{y}^{2}\right)$. Finally, the “sqrt” unit computes the depth in the scene, using Equation (9). In order to achieve high efficiency in the square root computation, we adapted the architecture developed by Yamin Li and Wanming Chu [38]. This architecture uses a shift register mechanism and compares the more significant/less significant bits to achieving the root square operation without using embedded multipliers.

## 5. Result and Discussion

The developed FPGA architecture was implemented in an FPGA Cyclone IV EP4CGX150CF23C8 of Altera. All modules were designed via Quartus II Web Edition version 10.1SP1, and all modules were validated via post-synthesis simulations performed in ModelSim Altera. For all tests, we consider k=3,s=2 (Equations (4) and (5)) since these values provided a relatively “good” performance for real world scenarios. In practice, we recommend these values as references. Higher k=3,s=2 values could provide higher accuracy, but processing speed and hardware requirements can be increased. On the other hand, lower k=3,s=2 values should provide higher performance in terms of hardware requirements/processing speed, but accuracy could decrease. The full hardware resource consumption of the architecture is shown in Table 1. Our algorithm formulation allows for a compact system design; it requires 66% of the total logic elements of the FPGA Cyclone IV EP4CGX150CF23C8. For memory bits, our architecture uses 74% of the total resources, which represents 26 block RAMs consumed mainly in the circular buffers. This hardware utilization enables one to target a relatively small FPGA device, so a small FPGA-based smart camera might be suitable for real-time embedded applications. In the following subsections, comparisons with previous work are presented. For optical flow, comparisons with previous FPGA-based optical flow algorithms are presented. For depth estimation, we presented a detailed discussion about the performance and limitations of the proposed algorithm compared with the current state of the art.

### 5.1. Performance for the Optical Flow Algorithm

In comparison with previous work, in Table 2, we present hardware resource utilization between our FPGA architecture and previous FPGA-based optical flow algorithms. There are several works [22,25,26,27] whose FPGA implementations aims to parallelize all recursive operations in the original mathematical formulation. Unfortunately, most popular formulations such as those based on KTL [28] or Horn-Schunck [33] have iterative operations that are hard to parallelize. As a result, most previous works have relatively high hardware occupancy/implementations compared with a full parallelizable design approach. Compared with previous works, our FPGA architecture outperforms most of those of previous works; for a similar image resolution, there are fewer logic elements and memory bits than those in Ref. [25,29] and fewer logic elements and memory bits than those of [27]. In Ref. [27], memory usage decreased by a multiscale coding, making it possible to store only half of the original image, but this reduction involves pixel interpolation for some cases, which increases the logic element usage. For Ref. [22], the authors introduced an iterative-parallel approach; this makes it possible to achieve low hardware requirements, but processing speed is low. Finally, for Ref. [26], a filtering-based approach made it possible to achieve low hardware requirements with relatively high accuracy and high processing speed, but the algorithmic formulation required the storage of several frames, requiring a large external memory (near 250 MB for store 3 entire frames), which increases the system size and cost.

In Table 3, speed processing for different image resolutions is shown. We synthesized different versions of our FPGA architecture (Figure 8), and we adapted the circular buffers in order to work with all tested image resolutions. We then carried out post-synthesis simulation in ModelSim Altera. In all cases, our FPGA architecture reached real-time processing. When compared with previous work (Table 4), our algorithm provided the highest speed processing and outperforms those of several previous works [22,25,26,27,29]. For HD images, our algorithm reaches real-time processing: more than 60 fps for a 1280 × 1024 image resolution.

**Figure 14:**
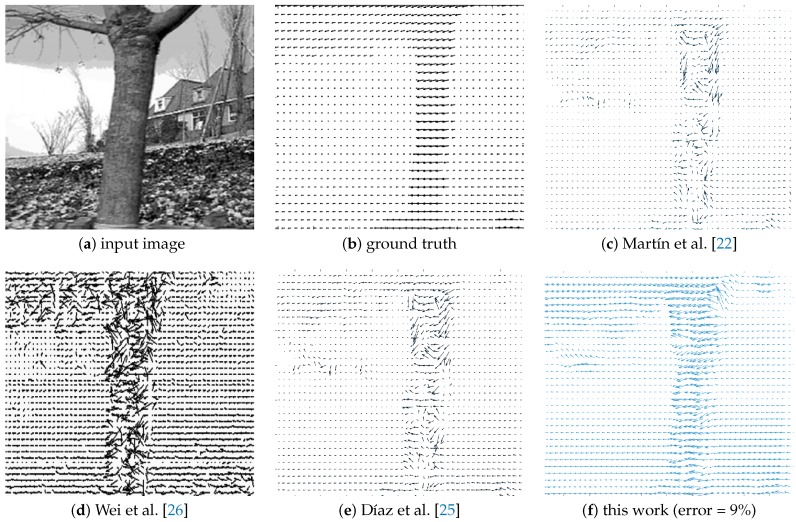
Accuracy performance for different FPGA-based optical flow algorithms.

**Figure 15:**
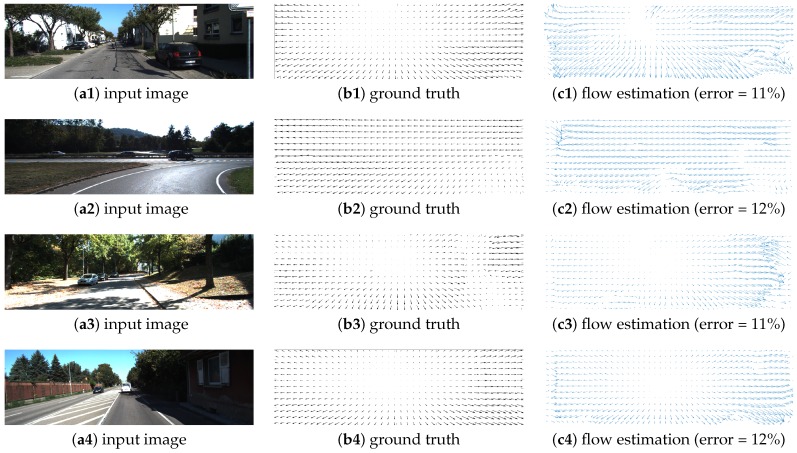
Optical flow: quantitative/qualitative results for the KITTI dataset.

In Figure 14, qualitative results for this work are shown alongside those of previous work. In a first experiment, we used the “Garden” dataset since others [22,25,26] have used this dataset as a reference. When compared with previous work (Figure 14), our algorithm shows a high performance under real world scenarios. It outperforms several previous works [22,25,26], quantitatively closer to the ground truth (error near to 9%) compared with other FPGA-based approaches. In a second experiment, quantitative and qualitative results for the KITTI dataset [35] are shown. In all cases, our algorithm provides high performance, reaching an error close to 10% with respect to several test sequences, as shown in Figure 15. In both experiments, we computed the error by comparing the ground truth $\Omega_{x}\left(x,y\right)$ and $\Omega_{y}\left(x,y\right)$ (provided with the dataset) with the computed optical flow $\Delta_{x}\left(x,y\right)$ and $\Delta_{y}\left(x,y\right)$. First, we computed the local error (the error magnitude at each point of the input image) as defined in Equation (10), where i,j is the input image resolution. A global error (Ξ) could then be computed as shown in Equation (11), where i,j is the input image resolution. ξ(x,y) is the local error at each pixel in the reference image, and the global error (Ξ) is the percentage of pixels in the reference image in which local error is closer to zero.

(10) $$\begin{array}{c} \xi \left(x,y\right)=\sum_{x=1}^{x=i}\sum_{y=1}^{y=j}\sqrt{\Omega_{x}{\left(x,y\right)}^{2}+\Omega_{y}{\left(x,y\right)}^{2}}-\sqrt{\Delta_{x}{\left(x,y\right)}^{2}+\Delta_{y}{\left(x,y\right)}^{2}} \end{array}$$

(11) $$\begin{array}{c} \Xi =\frac{100\%}{i\cdot j}\cdot \sum_{x=1}^{x=i}\sum_{y=1}^{y=j}\left\{\begin{array}{cc} 1 & if\;\xi (x,y)>=0\\ 0 & otherwise \end{array}\right.. \end{array}$$

### 5.2. Performance for the Depth Estimation Step

**Figure 16:**
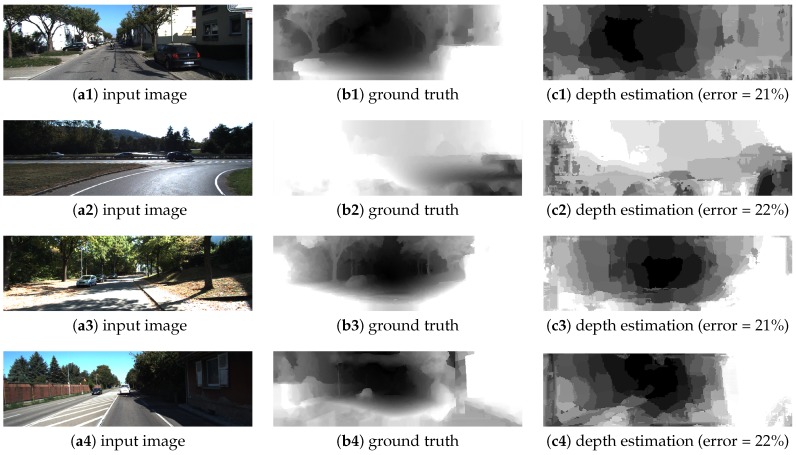
Depth estimation: quantitative/qualitative results for the KITTI dataset.

In Figure 16, quantitative and qualitative results for the KITTI dataset [35] are shown. In all cases, our algorithm provides rough depth maps compared with stereo-based or deep learning approaches [39,40] but with real-time processing and with the capability to be implemented in embedded hardware, suitable for smart cameras. To our knowledge, previous FPGA-based approaches are limited; there are several GPU-based approaches, but in these cases most of the effort was for accuracy improvements and real-time processing, or embedded capabilities were not considered; thus, in several cases details about the hardware requirements or the processing speed are not provided [41,42,43]. In Table 5, quantitative comparisons between our algorithm and the current state of the art can be made. For previous works, the RMS error, hardware specifications, and processing speed were obtained from the published manuscripts, while for our algorithm we computed the RMS error as indicated by the KITTI dataset [44]. For accuracy comparisons, most previous works [41,42,43,45,46,47] outperform our algorithm (near 15% more accurate than ours); however, our algorithm outperforms all of them in terms of processing speed (a processing speed up to 128 times faster than previous works) and with embedded capabilities (making it possible to develop a smart camera/sensor suitable for embedded applications).

**Figure 17:**
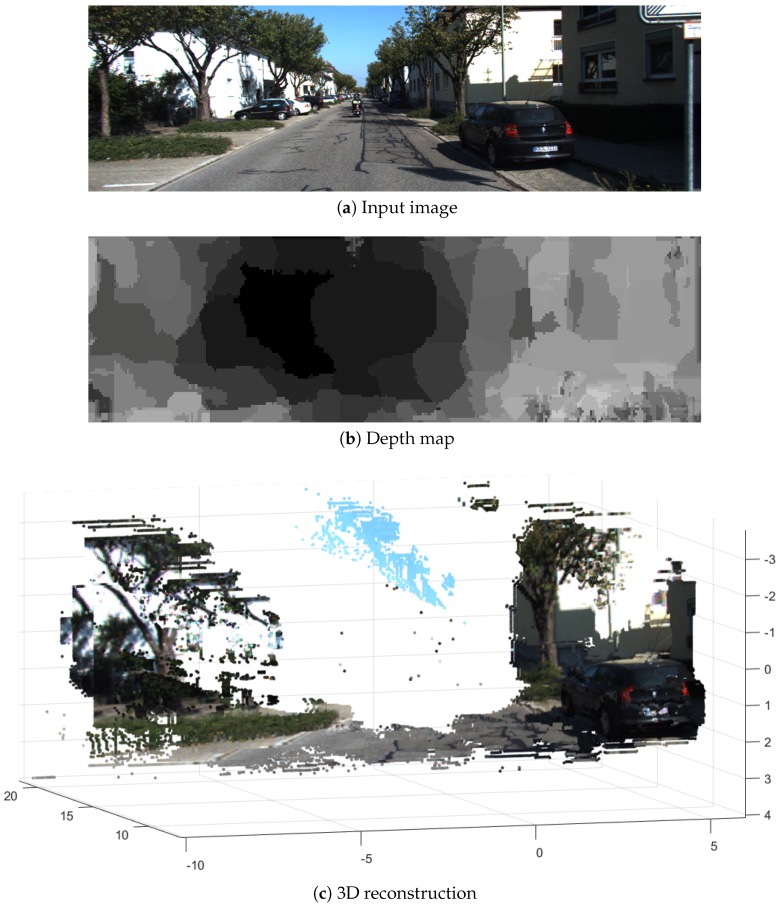
The KITTI dataset: Sequence 00; 3D reconstruction by the proposed approach. Our algorithm provides rough depth maps (a lower accuracy compared with previous algorithms) but with real-time processing and with the capability to be implemented in embedded hardware; as a result, real-time dense 3D reconstructions can be obtained, and these can be exploited by several real world applications such as augmented reality, robot vision and surveillance, and autonomous flying.

Finally, in Figure 17, an example of 3D reconstruction using our approach is shown. Our depth maps allow for a real-time dense 3D reconstruction. Previous works like the ORB-SLAM [48] or LSD-SLAM [49] compute motion and depth in 2–7% of all image pixels, while ours computes 80% of the image pixels. Thus, our algorithm improves by around 15 times the current state of the art, making possible real-time dense 3D reconstructions with the capability to be implemented inside FPGA devices, suitable for smart cameras.

## 6. Conclusions

Depth from motion is the problem of depth estimation using information from a single moving camera. Although several depth from motion algorithms have been developed, previous works have had low processing speeds and high hardware requirements that limit the embedded capabilities. In order to solve these limitations, we have proposed a new depth estimation algorithm whose FPGA implementation delivers high efficiency in terms of algorithmic parallelization. Unlike previous works, depth information is estimated in real time inside a compact FPGA device, making our mathematical formulation suitable for smart embedded applications.

Compared with the current state of the art, previous algorithms outperform our algorithm in terms of accuracy, but our algorithm outperforms all previous approaches in terms of processing speed and hardware requirements; these characteristics make our approach a promising solution for current embedded systems. We believed that several real world applications such as augmented reality, robot vision and surveillance, and autonomous flying can take advantage of our algorithm since it delivers real-time depth maps that can be exploited to create dense 3D reconstructions or other abstractions useful for extracting scene information.

## Figures and Tables

**Table 1:** Hardware resource consumption for the developed FPGA architecture.

Resource	Consumption/Image Resolution		
	640 × 480	320 × 240	256 × 256
Total logic elements	69,879 (59%)	37,059 (31%)	21,659 (18%)
Total pins	16 (3%)	16 (3%)	16 (3%)
Total Memory Bits	618,392 (15%)	163,122 (4%)	85,607 (2%)
Embedded multiplier elements	0 (0%)	0 (0%)	0 (0%)
Total PLLs	1 (25%)	1 (25%)	1 (25%)

**Table 2:** Hardware resource consumption.

Method	Logic Elements	Memory Bits	Image Resolution
Martín et al. [22] (2005)	11,520	147,456	256 × 256
Díaz et al. [25] (2006)	513,216	685,670	320 × 240
Wei et al. [26] (2007)	10,288	256 MB (DDR)	640 × 480
Barranco et al. [27] (2012)	82,526	573,440	640 × 480
Honegger et al. [29] (2012)	49,655	1,111,000	376 × 240
Our work *	69,879	624,244	640 × 480
Our work *	37,059	163,122	320 × 240
Our work *	21,659	85,607	256 × 256

* Operating frequency = 50 MHz.

**Table 3:** Processing speed for different image resolutions, operating frequency = 50 MHz.

Resolution	Frames/s	Pixels/s
1280 × 1024	68	90,129,200
640 × 480	297	91,238,400
320 × 240	1209	92,880,000
256 × 256	1417	92,876,430

**Table 4:** Processing speed comparisons.

Method	Resolution	Frames/s	Pixels/s
Martín et al. [22]	256 × 256	60	3,932,160
Díaz et al. [25]	320 × 240	30	2,304,000
Wei et al. [26]	640 × 480	64	19,550,800
Barranco et al. [27]	640 × 480	31	9,523,200
Honegger et al. [29]	376 × 240	127	11,460,480
Our work	640 × 480	297	91,238,400

**Table 5:** Depth estimation process in the literature: performance and limitations for the KITTI dataset.

Method	Error (RMS)	Speed	Image Resolution	Approach	
Zhou et al. [41] (2017)	6.8%	-	128 × 416	DfM-based *	-
Yang et al. [45] (2017)	6.5%	5 fps	128 × 416	CNN-based *	GTX 1080 (GPU)
Mahjourian et al. [46] (2018)	6.2%	100 fps	128 × 416	DfM-based *	Titan X (GPU)
Yang et al. [42] (2018)	6.2%	-	830 × 254	DfM-based *	Titan X (GPU)
Godard et al. [43] (2018)	5.6%	-	192 × 640	CNN-based *	-
Zou et al. [47] (2018)	5.6%	1.25 fps	576 × 160	DfM-based *	Tesla K80 (GPU)
Our work	21.5%	192 fps	1241 × 376	DfM-based *	Cyclone IV (FPGA)

* DfM: Depth from Motion, CNN: Convolutional Neural Network.
